# Relationship Between Leadership, Personality, and the Dark Triad in Workplace: A Systematic Review

**DOI:** 10.3390/bs15030297

**Published:** 2025-03-03

**Authors:** Carla Bueno-de la Fuente, Sandra Núñez-Rodríguez, Raquel de la Fuente-Anuncibay, Jerónimo J. González-Bernal

**Affiliations:** 1 Department of Education, University of Burgos, 09001 Burgos, Spain; cbd1006@alu.ubu.es (C.B.-d.l.F.); raquelfa@ubu.es (R.d.l.F.-A.); 2 Department of Health Sciences, University of Burgos, 09001 Burgos, Spain; jejavier@ubu.es

**Keywords:** Dark Triad, personality traits, job performance, job satisfaction, leadership

## Abstract

The objective of this systematic review was to explore the interaction between Dark Triad traits (narcissism, Machiavellianism, and psychopathy) and other personality dimensions in the workplace, and how these combinations impact the performance of employees and leaders. Fifteen empirical studies published since 2014 were analyzed, which assessed the influence of Dark Triad traits along with other personality dimensions, such as the traditional Big Five (neuroticism, extraversion, openness, agreeableness, and conscientiousness), on performance, interpersonal relationships, and organizational satisfaction. The results suggest that narcissism is negatively related to agreeableness and self-control, Machiavellianism to empathy and agreeableness, and psychopathy to conscientiousness and empathy. These traits also affect job performance, as narcissism and Machiavellianism are associated with work addiction, while psychopathy is linked to lower responsibility and self-control. Additionally, leaders with high levels of Dark Triad traits experience reduced performance, especially in lower hierarchical roles. It was found that emotional stability can moderate the negative impact of narcissism, and that less regulated organizational contexts exhibit more interactions involving these dark traits. These findings provide valuable insights for developing talent assessment and management strategies aimed at improving organizational performance and climate through evidence-based approaches.

## 1. Introduction

The influence of personality in the workplace is a widely researched topic in the field of organizational psychology. The interaction between different personality traits not only affects individual behavior but also shapes how organizations operate and achieve their objectives. In this context, certain combinations of personality traits have been shown to significantly impact team performance and dynamics. Recently, interest in the Dark Triad of personality—comprising narcissism, Machiavellianism, and psychopathy—has grown, as these traits have particular characteristics that, depending on the environment and their interaction with other personality aspects, can influence job performance in positive or negative ways (Aluja et al., 2022; Doerfler et al., 2021; Gómez-Leal et al., 2023; Kaufman et al., 2019).

Traditionally, Dark Triad traits have been associated with negative behaviors, such as manipulation, lack of empathy, and exploiting others for personal gain (Aluja et al., 2022; Doerfler et al., 2021; Duradoni et al., 2023; Junça-Silva & Silva, 2023; Nguyen et al., 2021). These traits, however, do not always lead to harmful outcomes in the workplace. In certain situations, individuals with high levels of some of these traits can exhibit strategic advantages, especially in highly competitive environments or those requiring quick decision-making (Kaufmann et al., 2021; McLarty & Holt, 2019; Nguyen et al., 2021). In this sense, narcissism can be associated with high levels of confidence and charisma—qualities that, in leadership contexts, can be beneficial if moderated by other personality traits that promote cooperation and ethics (Brunzel, 2021; Fatfouta, 2019).

The current literature suggests that the effects of the Dark Triad on job performance are not independent of other personality traits (Aluja et al., 2022; Miao et al., 2019). Among the most influential theories is the Big Five model, which describes five fundamental dimensions of personality: openness to experience, conscientiousness, extraversion, agreeableness, and emotional stability. (Alderotti et al., 2023). These traits have traditionally been linked to positive outcomes in the workplace, such as performance, satisfaction, and adaptability to change (Alderotti et al., 2023; Hadziahmetovic & Mujezinovic, 2021; Kariyawasam & Welmilla, 2020; Putro & Tirtoprojo, 2022). The interaction between these traits can enhance or mitigate the effects of the Dark Triad at work. For example, a high degree of conscientiousness might moderate Machiavellian impulses, allowing for a calculated approach that does not compromise organizational ethics. This more nuanced approach helps to understand how certain combinations of traits can lead to exceptional performance or, in some cases, to problematic behaviors that impact both individual and collective well-being (Ellen et al., 2021; Kowalski et al., 2021; Musek & Grum, 2021; Rico-Bordera et al., 2023).

Likewise, other personality traits, such as humility or kindness, help buffer the darker personality traits. Though not included in the Big Five model, these traits have shown a significant role in moderating narcissistic, Machiavellian, or psychopathic tendencies, promoting ethical, collaborative, and empathetic behaviors in the workplace (Howard & Van Zandt, 2020; Hudson, 2023; Puthillam et al., 2020).

This systematic review aims to explore the interaction between Dark Triad traits and other dimensions of personality in the workplace, addressing how these combinations impact the performance of workers and leaders. To achieve this, empirical studies will be analyzed that examine both the presence of these traits in workers and their influence on performance, interpersonal relationships, and organizational satisfaction. The results of this review will contribute to the development of advanced strategies for talent evaluation and management, aimed at optimizing organizational performance. These findings will enable human resources professionals, managers, and organizational psychologists to implement evidence-based approaches to improve labor effectiveness, strengthen the organizational climate, and mitigate the impact of dysfunctional behaviors in the workplace.

A key objective of this review is to address the broader implications of these findings for organizational psychology and human resource management. By synthesizing existing research, this study aims to answer the following guiding question: How do Dark Triad traits interact with other personality dimensions in the workplace, and what are their consequences for individual and organizational outcomes? Additionally, sub-questions include the following: What research trends can be identified in this area? and How has the perception and study of the Dark Triad evolved over time?

By clarifying these aspects, this review contributes to the development of advanced talent evaluation and management strategies that optimize organizational performance. The findings will provide insights for human resources professionals, managers, and organizational psychologists, facilitating the implementation of evidence-based approaches to enhance workplace effectiveness, strengthen organizational climate, and mitigate the impact of dysfunctional behaviors. Furthermore, by identifying gaps in the literature and proposing future research directions, this review aims to foster a more nuanced understanding of the Dark Triad in the workplace.

## 2. Materials and Methods

This systematic review was conducted following the recommendations of the PRISMA Statement (Urrútia & Bonfill, 2010), as well as the research protocol developed. First, the search began with the formulation of a research question using the PIO format (Table 1), following the criteria established by Sackett et al.

Once the question was established, the systematic literature review was conducted between 11 and 21 October 2024 by consulting the electronic versions of the Scopus, Web of Science, ScienceDirect, and PubMed databases. Appropriate “Medical Subject Headings” (MeSH) were used, combined with Boolean operators (AND/OR) and free-text terms, some of which were truncated, in order to cover all possible variations of endings (Table 2).

The inclusion criteria considered for the selection of studies were as follows: original research examining adult workers in a workplace setting, regardless of the type of organization, industry, or sector. Additionally, the studies had to specifically assess the Dark Triad personality traits in the workplace, such as narcissism, Machiavellianism, and subclinical psychopathy, as well as the five major personality dimensions (Neuroticism, Extraversion, Openness, Agreeableness, and Conscientiousness). Studies that addressed leadership and explored its relationship with personality dimensions in the context of the Dark Triad were prioritized. Descriptive studies with a cross-sectional or longitudinal design, published from 2014 onwards, in English or Spanish, were accepted.

On the other hand, the exclusion criteria included studies focused on non-work populations, such as students or unemployed individuals, as well as those that did not address or use the components of the Dark Triad. Also excluded were studies that did not provide clear comparisons between leadership and personality traits, as well as non-empirical research such as reviews, case studies, editorials, opinions, or commentaries.

As a complementary strategy, a manual backward search, also known as “snowball sampling”, was conducted to identify relevant studies that may have been initially overlooked. Additionally, sources of documentation and grey literature, with less coverage and access, as well as the bibliographic references included in the selected studies, were reviewed.

On the other hand, the selection of studies and the assessment of their methodological quality were carried out by pairs of reviewers, independently and blind. Any discrepancies were resolved by consensus, or if necessary, with the intervention of a third evaluator. To ensure consistency among the researchers in data collection, a standardized information extraction form was created, which included the following elements for each selected article: title and lead author, country and year of publication, study type and objective, study location and period, sample size and characteristics, definition of variables and instruments used, a summary of the results obtained and conclusions, as well as the evaluation of its scientific and technical quality. Methodological quality and risk of bias were assessed using the “critical appraisal tools” from the Joanna Briggs Institute at the University of Adelaide (Australia) (Jordan et al., 2019), adjusted to the design of each study (Moola et al., 2020). A minimum threshold of 6 out of 8 points was established for cross-sectional descriptive studies as an inclusion criterion in the systematic review. Additionally, a pilot test was conducted in which each reviewer evaluated 3 articles, and then the agreement between their assessments was analyzed.

## 3. Results

**Figure 1:**
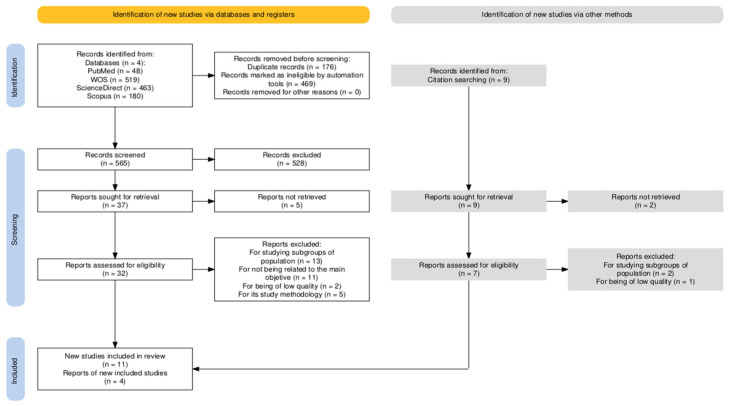
Study selection flowchart.

A total of 1214 studies were initially identified, of which 15 were selected for this review after conducting a full-text critical reading, following the PRISMA screening guidelines (Figure 1).

Appendix A, the main characteristics and findings of the selected studies can be seen.

### 3.1. Characteristics of the Studies

The number of participants in the studies included in this review ranged from 53 to 6957, with a total of 16,621 individuals, with a predominance of male participants in most studies. The participants were primarily adult workers, with ages ranging from 18 to 70 years old. These studies focused on analyzing the effects of dark personality traits, such as Machiavellianism, narcissism, and psychopathy, as well as exploring other personality traits, across different dimensions of job performance and job satisfaction, in order to understand the relationship between personality traits and performance or organizational behavior.

Regarding the study designs, all studies (n = 15) had a descriptive cross-sectional design. The geographic distribution suggests a variety of regions and countries with different work contexts, including sectors such as political and private sectors: Turkey (n = 1), UK (n = 3), Canada (n = 3), USA (n = 5), China (n = 1), Germany (n = 1), Spain (n = 1), and Australia (n = 1). Some study samples were recruited from different countries. Furthermore, the studies included in this review analyze different managerial and leadership positions.

To measure dark personality traits, different scales were used: the Dark Triad Scale (SD3) (n = 3), along with other specific instruments such as the Machiavellianism IV (n = 2) and the Dirty Dozen (n = 2). In some cases, the dark triad traits were measured separately, using instruments like the LSRP for psychopathy (n = 2), the NPI-16 and NPI-R narcissism scales (n = 2), and the HDS (n = 3), the EPA psychopathy scale (n = 1), and the FFNI for narcissism (n = 1). The Big Five traits were predominantly assessed with the NEO-PI-R (n = 4), although other studies used the Mini-IPIP (n = 1) or the NEO-FFI (n = 2). Additionally, several studies used questionnaires like the PPQ (n = 1) and PSI (n = 1) to measure political skills and competencies. The dimensions of job performance were mainly evaluated with individual job performance questionnaires (n = 2), the CWB (n = 1), OCB (n = 1), and the multifactor leadership questionnaire (n = 1); while work engagement and job satisfaction were measured with the UWES (n = 1) and the Fahrenberg Life Satisfaction questionnaire (n = 1), respectively.

In terms of statistical analysis, most of the studies applied univariate and multivariate tests to examine the relationship between dark and bright personality traits and work outcomes. Studies that analyzed leadership factors or adaptive performance also employed trajectory analysis models.

Regarding methodological quality and risk of bias, most of the studies received satisfactory scores based on the evaluated criteria (Table 3), meeting the established methodological standards.

Methodological quality and risk of bias were assessed using the “critical appraisal tools” from the Joanna Briggs Institute at the University of Adelaide (Australia) (Jordan et al., 2019), adjusted to the design of the studies (Moola et al., 2020). A minimum threshold of 6 out of 8 points was established for cross-sectional descriptive studies as an inclusion criterion in the systematic review. This threshold was chosen to ensure that included studies met a baseline level of methodological rigor, reducing the risk of bias and enhancing the reliability of the findings. Studies scoring below this level were considered to have methodological weaknesses that could compromise the validity of their results.

### 3.2. Description of the Results

All the studies included in this systematic review examine the relationship between the Dark Triad traits (narcissism, Machiavellianism, and psychopathy) and other personality dimensions. Most of them measure the Big Five dimensions in the workplace context (DeShong et al., 2017; Fernández-del-Río et al., 2020; Furnham & Crump, 2014; Grover & Furnham, 2021; Kızıloğlu et al., 2024; McKee et al., 2017; Paleczek et al., 2018; Ramos-Villagrasa et al., 2020; Simonet et al., 2018), as well as the impact of these interactions on the job performance of workers and leaders. Other studies also examine additional personality traits (Furnham & Treglown, 2021; Harrell et al., 2024; Schattke & Marion-Jetten, 2022; Shi et al., 2024; Wiens & Walker, 2019). It is conducted in different contexts, measuring a variety of consequences of the interaction of these traits in the workplace.

The findings on the Dark Triad, which includes narcissism, Machiavellianism, and psychopathy, reveal significant associations both among themselves and with the Big Five personality traits: neuroticism, extraversion, openness, agreeableness, and conscientiousness (DeShong et al., 2017; Furnham & Crump, 2014; Harrell et al., 2024; Kızıloğlu et al., 2024; McKee et al., 2017; Paleczek et al., 2018; Ramos-Villagrasa et al., 2020; Simonet et al., 2018; Wiens & Walker, 2019). In particular, narcissism is negatively related to agreeableness, self-control, and neuroticism, suggesting that individuals with high levels of narcissism tend to show less empathy and emotional regulation (Furnham & Crump, 2014; Harrell et al., 2024; Ramos-Villagrasa et al., 2020; Wiens & Walker, 2019). On the other hand, Machiavellianism shows negative correlations with agreeableness, conscientiousness, and empathy, indicating that those who exhibit this trait tend to be less trustworthy and less compassionate (DeShong et al., 2017; Harrell et al., 2024; Kızıloğlu et al., 2024; McKee et al., 2017; Paleczek et al., 2018; Ramos-Villagrasa et al., 2020; Wiens & Walker, 2019). Regarding psychopathy, it was observed that it negatively correlates with responsibility, empathy, and self-control (DeShong et al., 2017; Harrell et al., 2024; Ramos-Villagrasa et al., 2020; Wiens & Walker, 2019), which implies that individuals with psychopathic traits often struggle to maintain responsible and empathetic behaviors.

The analysis of demographic differences reveals that women tend to exhibit higher levels of agreeableness compared to men in workplace environments. Additionally, women show higher scores in traits such as sincerity, altruism, anxiety, and vulnerability (DeShong et al., 2017; Grover & Furnham, 2021; McKee et al., 2017; Paleczek et al., 2018; Ramos-Villagrasa et al., 2020; Wiens & Walker, 2019). In contrast, men scored higher in traits of the Dark Triad, including Machiavellianism, psychopathy, and desire for control (DeShong et al., 2017; Grover & Furnham, 2021).

Regarding job performance, narcissism and Machiavellianism are positively related to workaholism, along with extraversion and neuroticism (Kızıloğlu et al., 2024), and are negatively correlated with leadership performance (Ramos-Villagrasa et al., 2020; Simonet et al., 2018), suggesting that these traits may promote dark leadership behaviors. Despite this, narcissism is positively associated with leadership positions and higher salaries (Paleczek et al., 2018; Schattke & Marion-Jetten, 2022). Machiavellianism and psychopathy, which are highly correlated with each other, are negatively associated with self-control, responsibility, empathy, agreeableness, and, considering the neuroticism trait, with resilience and analytical skills (DeShong et al., 2017; Paleczek et al., 2018; Ramos-Villagrasa et al., 2020; Silvester et al., 2014; Wiens & Walker, 2019), indicating that individuals with high levels of these traits may have reduced adaptive job performance.

In this way, it has been shown that leaders with high levels of narcissism, Machiavellianism, and psychopathy tend to experience a decline in their performance, particularly in lower hierarchical roles (Harrell et al., 2024; Kızıloğlu et al., 2024; Paleczek et al., 2018; Ramos-Villagrasa et al., 2020; Shi et al., 2024; Simonet et al., 2018; Wiens & Walker, 2019). In one study, it was observed that leaders with high levels of narcissism combined with Machiavellian tendencies had significantly lower performance compared to those who only exhibited some dark traits (Simonet et al., 2018). Additionally, the combination of narcissism and histrionic traits, which seek excessive attention, also had a negative impact on performance (Simonet et al., 2018). Emotional stability, on the other hand, emerges as an important moderating factor in the relationship between narcissism and job performance, buffering its negative effect (Harrell et al., 2024; Ramos-Villagrasa et al., 2020; Wiens & Walker, 2019). It is observed that leaders with high levels of narcissism and low emotional stability show significantly lower performance (Harrell et al., 2024; Ramos-Villagrasa et al., 2020; Wiens & Walker, 2019). Additionally, organizational contexts play an important role, with interactions of dark personality traits being more evident in less regulated sectors, compared to highly regulated contexts (Paleczek et al., 2018; Shi et al., 2024; Simonet et al., 2018).

The traits of the Dark Triad also predict a preference for leaders who exhibit dark behaviors, regardless of demographic factors. It is confirmed that agreeableness and conscientiousness are negatively correlated with the desire for such leadership behaviors, while the Dark Triad accounts for a significant portion of the variability in preference for dark leaders, suggesting that these traits are influential in leadership decisions (DeShong et al., 2017; Harrell et al., 2024; Kızıloğlu et al., 2024; McKee et al., 2017; Paleczek et al., 2018; Ramos-Villagrasa et al., 2020; Shi et al., 2024; Silvester et al., 2014; Simonet et al., 2018).

While most of the analyzed studies highlight the negative effects of the Dark Triad in the workplace, some findings suggest that certain traits can provide strategic advantages in specific contexts. For example, narcissism is associated with greater self-confidence and persuasive skills, which can benefit leadership and decision-making in highly competitive environments (Kaufmann et al., 2021; Kızıloğlu et al., 2024; McLarty & Holt, 2019; Nguyen et al., 2021; Paleczek et al., 2018; Ramos-Villagrasa et al., 2020; Schattke & Marion-Jetten, 2022; Silvester et al., 2014). Similarly, Machiavellianism, when combined with high levels of emotional intelligence and ethical regulation, can promote effective strategic management, enhancing negotiation and conflict resolution (2–5). Additionally, in scenarios that require quick decision-making and adaptation to uncertainty, the lower risk aversion associated with psychopathy facilitates agile and effective responses (5,6).

In summary, the findings of this systematic review highlight the complexity of the relationships between the Dark Triad traits and the Big Five in workplace contexts. Dark traits not only influence performance and leadership preferences but also interact with demographic and contextual variables that affect their manifestation in work environments.

## 4. Discussion

From a general perspective, the aim of this systematic review was to explore and synthesize the relationship between dark personality traits and other dimensions of personality in the workplace, focusing on how these traits influence various aspects of performance, leadership, preferences in supervisory styles, and work adaptation.

To facilitate a clearer interpretation of the results, we present the following table, which synthesizes key aspects of the discussion by linking past research, current findings, and their implications for future trends and practical applications (Table 4):

The results reveal consistent associations between the Dark Triad and the Big Five, particularly through negative correlations with agreeableness, conscientiousness, and emotional stability (DeShong et al., 2017; Harrell et al., 2024; McKee et al., 2017; Paleczek et al., 2018; Ramos-Villagrasa et al., 2020; Wiens & Walker, 2019). For example, individuals with high levels of narcissism exhibit negative interaction patterns by showing less empathy and emotional control, which affects their ability to cooperate and collaborate (Kızıloğlu et al., 2024; Paleczek et al., 2018; Shi et al., 2024; Simonet et al., 2018; Wiens & Walker, 2019). These findings are consistent with previous studies that associate narcissism with an inflated self-perception that minimizes receptivity and empathy towards others, as well as Machiavellianism, which is characterized by a lack of trust and empathy (Aluja et al., 2022; Doerfler et al., 2021; Duradoni et al., 2023; Junça-Silva & Silva, 2023; Nguyen et al., 2021). his can be interpreted as a mechanism that fosters instrumental relationships in which manipulation and self-interest prevail over genuine collaboration. Finally, psychopathy shows negative correlations with responsibility and self-control (DeShong et al., 2017; Harrell et al., 2024; Paleczek et al., 2018; Wiens & Walker, 2019), suggesting that individuals with psychopathic traits may be less committed to organizational norms, prioritizing their own needs over ethics and respect for rules. These patterns show that the Dark Triad not only affects the quality of individual interactions but also contributes to the creation of a less cohesive and cooperative organizational climate, which can lead to issues with talent retention and collective performance (Aluja et al., 2022; DeShong et al., 2017; Doerfler et al., 2021; Duradoni et al., 2023; Ellen et al., 2021; Gómez-Leal et al., 2023; Grover & Furnham, 2021; Howard & Van Zandt, 2020; Hudson, 2023; Junça-Silva & Silva, 2023; Kızıloğlu et al., 2024; McKee et al., 2017; Musek & Grum, 2021; Nguyen et al., 2021; Paleczek et al., 2018; Puthillam et al., 2020; Rico-Bordera et al., 2023; Simonet et al., 2018; Wiens & Walker, 2019).

Narcissism, although associated with favorable economic outcomes such as higher salaries and the attainment of leadership positions, appears to have a dual impact on performance. While some studies suggest that narcissism can enhance self-confidence and strategic vision in leadership roles (Kaufmann et al., 2021; Kızıloğlu et al., 2024; McLarty & Holt, 2019; Nguyen et al., 2021; Paleczek et al., 2018; Ramos-Villagrasa et al., 2020; Schattke & Marion-Jetten, 2022; Silvester et al., 2014), it can also lead to egocentric and domineering behaviors that harm teamwork and limit the adaptive potential of the leader (Brunzel, 2021; Fatfouta, 2019; Paleczek et al., 2018; Ramos-Villagrasa et al., 2020; Schattke & Marion-Jetten, 2022; Simonet et al., 2018; Wiens & Walker, 2019). This phenomenon is especially notable when narcissism is combined with low emotional stability, an interaction that tends to amplify its negative effects (Aluja et al., 2022; Miao et al., 2019; Schattke & Marion-Jetten, 2022; Shi et al., 2024; Simonet et al., 2018). Narcissistic leaders with low emotional stability, for example, underperform, implying that lack of emotional self-regulation compounds their difficulty in managing the complexities of organizational responsibility.

As for Machiavellianism and psychopathy, both traits are negatively correlated with adaptive performance and self-control, and affect resilience and analytical skills (Ellen et al., 2021; Hadziahmetovic & Mujezinovic, 2021; Kızıloğlu et al., 2024; Kowalski et al., 2021; Ramos-Villagrasa et al., 2020; Rico-Bordera et al., 2023; Silvester et al., 2014; Simonet et al., 2018; Wiens & Walker, 2019). This finding indicates that individuals with high levels of these traits not only underperform in leadership roles but also lack the competencies necessary to adapt to the changing demands of the work environment. Such reduced adaptability represents a risk in positions that require effective and ethical decision-making, as Machiavellianism and psychopathy foster exploitative behavior and abuse of power (DeShong et al., 2017; Furnham & Treglown, 2021; Kızıloğlu et al., 2024; Kowalski et al., 2021; Musek & Grum, 2021; Nguyen et al., 2021; Paleczek et al., 2018; Ramos-Villagrasa et al., 2020; Simonet et al., 2018; Wiens & Walker, 2019).

This review also highlights the importance of demographic and contextual variables in the manifestation of these traits. In general, women were found to have higher levels of agreeableness and conscientiousness, while men tended to score higher on Dark Triad traits, especially Machiavellianism and psychopathy (Chiorri et al., 2019; DeShong et al., 2017; Grover & Furnham, 2021; Jonason & Davis, 2018; Lee et al., 2013; Paleczek et al., 2018). These results suggest that the gender composition of a team may influence power dynamics and collaboration, and that in highly competitive work contexts, dark traits in male leaders may be perceived as strengths, especially in less regulated sectors (Chiorri et al., 2019; Kaufmann et al., 2021; Lee et al., 2013; Nguyen et al., 2021).

Another relevant aspect is the association between the Dark Triad and the preference for leaders with dark behaviors. The review confirms that employees high in these traits tend to prefer and support leaders who display dominance and manipulative behaviors (McKee et al., 2017; Schattke & Marion-Jetten, 2022; Simonet et al., 2018). This preference may create an organisational culture that reinforces dysfunctional behaviors, diminishing the organization’s attractiveness to employees who value ethics, respect and collaboration (Aluja et al., 2022; Gómez-Leal et al., 2023; McLarty & Holt, 2019; Nguyen et al., 2021). The negative relationship between kindness and conscientiousness with preference for dark leadership indicates that these ‘prosocial’ traits may act as barriers to the acceptance of abusive or manipulative leadership, suggesting the need to foster these traits in the organizational culture to counteract the negative influences of the Dark Triad (7,8).

The results also indicate that, although narcissism may be positively related to the attainment of leadership positions and higher salaries, the combination of this trait with low emotional stability and Machiavellianism tends to impair performance and effectiveness in leadership positions (Fernández-del-Río et al., 2020; Paleczek et al., 2018; Ramos-Villagrasa et al., 2020; Simonet et al., 2018; Wiens & Walker, 2019). This is consistent with literature suggesting that dark traits can facilitate ‘dark leadership,’ characterized by the exploitation and manipulation of subordinates, leading to a less healthy and productive work environment (Brunzel, 2021; Fatfouta, 2019; Hudson, 2023; Kaufmann et al., 2021; Nguyen et al., 2021). Furthermore, the finding that narcissism and Machiavellianism are related to workaholism underscores that while these traits may motivate high work engagement, their effect on overall performance and leadership sustainability tends to be negative (Aluja et al., 2022; Ellen et al., 2021; Furnham & Treglown, 2021; Kızıloğlu et al., 2024; Paleczek et al., 2018; Simonet et al., 2018; Wiens & Walker, 2019).

The review also highlights the roles of emotional stability and the Big Five as a key moderator in the relationship between narcissism and job performance (Grover & Furnham, 2021; Paleczek et al., 2018; Schattke & Marion-Jetten, 2022; Simonet et al., 2018). For example, leaders with high emotional stability, humility or emotional intelligence appear to buffer the negative impact of their own narcissism, while those with low levels of narcissism exhibit significantly lower performance, which is consistent with models suggesting that emotional regulation facilitates greater adaptability and control in stressful situations (Aluja et al., 2022; Grover & Furnham, 2021; Howard & Van Zandt, 2020; Kowalski et al., 2021; Miao et al., 2019; Paleczek et al., 2018; Rico-Bordera et al., 2023; Schattke & Marion-Jetten, 2022; Simonet et al., 2018). Furthermore, in line with the existing literature, the findings indicate that organizational sector influences the manifestation of dark traits, with less regulated sectors showing a higher prevalence of dark leadership behaviors compared to regulated sectors, which could suggest that certain environments encourage and enable less controlled and more abusive leadership (Brunzel, 2021; Cohen, 2016; McKee et al., 2017; McLarty & Holt, 2019; Paleczek et al., 2018; Palmer et al., 2017; Shi et al., 2024; Simonet et al., 2018).

Another relevant aspect is the association between the Dark Triad and the preference for leaders with dark behaviors. The review confirms that employees high in these traits tend to prefer and support leaders who display dominance and manipulative behaviors (McKee et al., 2017; Paleczek et al., 2018; Schattke & Marion-Jetten, 2022). This preference could create an organizational culture that reinforces dysfunctional behaviors, diminishing the attractiveness of the organization to employees who value ethics, respect and collaboration (Aluja et al., 2022; Furnham & Treglown, 2021; Nguyen et al., 2021; Wiens & Walker, 2019). The negative relationship between kindness and conscientiousness with preference for dark leadership indicates that these ‘prosocial’ traits may act as barriers to the acceptance of abusive or manipulative leadership, suggesting the need to foster these traits in the organizational culture to counteract the negative influences of the Dark Triad (Brunzel, 2021; DeShong et al., 2017; Fatfouta, 2019; Fernández-del-Río et al., 2020; Furnham & Treglown, 2021; McKee et al., 2017; Schattke & Marion-Jetten, 2022; Simonet et al., 2018).

The heterogeneity of the studies included in this systematic review represents a significant limitation, as variations in organizational contexts, measures used and populations studied make it difficult to compare and generalize findings. In addition, the predominance of cross-sectional studies limits our understanding of personality dynamics, preventing us from assessing their evolution and lasting effects on job performance. The research procedure used also restricts the ability to infer causal relationships, as most studies rely on correlational designs rather than experimental or longitudinal approaches. Furthermore, the lack of meta-analyses in this area hinders the ability to synthesize findings across studies systematically, limiting the robustness of the conclusions drawn. Finally, there is a possible publication bias, as studies tend to report negative effects of the Dark Triad more frequently than neutral or positive effects. This bias might have influenced the general interpretation of the findings and the perception of these traits as exclusively dysfunctional, without considering possible positive impacts under certain contexts or combinations with other traits.

In contrast, these findings present several important implications for professional practice in human resource management, organizational development and leadership. Understanding the interaction between Dark Triad traits and personality dimensions allows for the development of more effective assessment and selection strategies aimed at identifying and managing talent that not only optimize job performance, but also foster a healthy work environment. Furthermore, these findings can guide the implementation of personal and professional development programs that mitigate the negative effects of dark traits on leadership and performance, promoting an organizational culture based on empathy, collaboration and self-control.

Future research directions should therefore focus on addressing the limitations identified in this review, particularly the heterogeneity of results and the paucity of longitudinal studies. Research using longitudinal designs is essential to capture the evolution of Dark Triad traits and their impact on job performance over time. In addition, it is recommended to explore the influence of diverse cultural and organizational contexts on the relationship between these traits and personality dimensions, as well as their effect on leadership. Finally, investigating interventions that may moderate or mitigate the negative effects of Dark Triad traits in the work environment could provide valuable information for improving organizational dynamics and employee well-being.

## 5. Conclusions

This systematic review has provided a comprehensive overview of the relationship between Dark Triad traits (narcissism, Machiavellianism and psychopathy) and the Big Five personality dimensions in the workplace. The findings suggest that these dark traits not only have a significant impact on work performance, leadership behavior, and interpersonal dynamics, with emotional stability acting as a key moderating factor.

Theoretically, this study contributes to understanding how the Dark Triad interacts with other personality traits, but further research is needed to develop models that incorporate contextual factors such as organizational culture and industry-specific variables. Methodologically, while the review identifies consistent trends, there are opportunities to further standardize measurement tools and adopt longitudinal studies to assess the long-term impact of the Dark Triad traits on career development and team dynamics. This approach could lead to more comprehensive and generalizable findings across diverse organizational environments. Practically, the findings suggest the importance of integrating Dark Triad traits into recruitment and leadership development strategies. HR professionals can use psychometric tools to assess these traits, and organizations should focus on fostering emotional stability and ethical leadership to mitigate their negative effects.

Future research could explore the potential positive aspects of these traits in competitive or high-pressure contexts and examine their impact on emerging workplace challenges, such as remote work and the growing emphasis on emotional intelligence in leadership.

## Figures and Tables

**Table 1:** PIO Format.

Population	Workers in a Workplace Setting
Intervention	Interaction between Dark Triad traits and the Big Five personality dimensions
Outcomes	Effects of the interaction between Dark Triad traits and other personality dimensions on job performance.
Research Question	How are Dark Triad personality traits related to other personality dimensions in workers and leaders, and how do these interactions affect job performance?

**Table 2:** Search strategy used, adapted to each of the databases.

Database	Search Strategy
Pubmed	((“Work” OR “occupational setting” OR “organization*”) AND (“Dark Triad” OR “narcissis*” OR “Machiavell*” OR “psychopath*”) AND (“Leadership” OR “leadership style*” OR “transformational” OR “authoritarian” OR “democratic” OR “charismatic”) AND (“Personality” OR “Big Five” OR “NEO-PI-3” OR “Neuroticism” OR “Extraversion” OR “Openness” OR “Agreeableness” OR “Conscientiousness”))
Web of Science	TS = (“Work” OR “occupational setting” OR “organization*”) AND TS = (“Dark Triad” OR “narcissis*” OR “Machiavell*” OR “psychopath*”) AND TS = (“Leadership” OR “leadership style*” OR “transformational” OR “authoritarian” OR “democratic” OR “charismatic”) AND TS = (“Personality” OR “Big Five” OR “NEO-PI-3” OR “Neuroticism” OR “Extraversion” OR “Openness” OR “Agreeableness” OR “Conscientiousness”)
Scopus	TITLE-ABS-KEY(“Work” OR “occupational setting” OR “organization*”) AND TITLE-ABS-KEY(“Dark Triad” OR “narcissis*” OR “Machiavell*” OR “psychopath*”) AND TITLE-ABS-KEY(“Leadership” OR “leadership style*” OR “transformational” OR “authoritarian” OR “democratic” OR “charismatic”) AND TITLE-ABS-KEY(“Personality” OR “Big Five” OR “NEO-PI-3” OR “Neuroticism” OR “Extraversion” OR “Openness” OR “Agreeableness” OR “Conscientiousness”)
Science Direct	(“Dark Triad” OR “narcissism” OR “Machiavellianism” OR “psychopathy”) AND (“Leadership” OR “transformational” OR “authoritarian”) AND (“Personality traits” OR “NEO-PI-3” OR “Big Five”) AND “workplace”

**Table 3:** Results of the quality assessment of descriptive studies.

Study	JBI	Q1	Q2	Q3	Q4	Q5	Q6	Q7	Q8
(Kızıloğlu et al., 2024)	7/8	+	+	+	+	-	+	+	+
(Silvester et al., 2014)	8/8	+	+	+	+	+	+	+	+
(Simonet et al., 2018)	8/8	+	+	+	+	+	+	+	+
(McKee et al., 2017)	8/8	+	+	+	+	+	+	+	+
(Grover & Furnham, 2021)	8/8	+	+	+	+	+	+	+	+
(DeShong et al., 2017)	6/8	+	+	+	+	-	-	+	+
(Wiens & Walker, 2019)	6/8	+	+	+	+	-	-	+	+
(Harrell et al., 2024)	7/8	+	+	+	+	+	-	+	+
(Shi et al., 2024)	8/8	+	+	+	+	+	+	+	+
(Schattke & Marion-Jetten, 2022)	6/8	+	+	+	+	-	-	+	+
(Furnham & Crump, 2014)	8/8	+	+	+	+	+	+	+	+
(Paleczek et al., 2018)	8/8	+	+	+	+	+	+	+	+
(Ramos-Villagrasa et al., 2020)	8/8	+	+	+	+	+	+	+	+
(Fernández-del-Río et al., 2020)	8/8	+	+	+	+	+	+	+	+
(Furnham & Treglown, 2021)	7/8	+	+	+	+	+	-	+	+

**Table 4:** Key aspects of the discussion.

Aspect	Description
Antecedents	Previous literature has established that Dark Triad traits influence workplace dynamics, often associating them with negative behaviors such as manipulation, low empathy, and leadership difficulties. However, some studies suggest potential strategic advantages in competitive environments.
Current Findings	Our systematic review supports previous research by confirming that Dark Triad traits are significantly associated with workplace outcomes, including job performance, leadership effectiveness, and interpersonal relationships. However, the findings also highlight that these effects are not uniform across all contexts. Factors such as organizational structure, regulatory environments, and emotional stability appear to moderate the influence of these traits.
Trends	The findings suggest the need to improve HR evaluation tools, such as psychometric tests, to identify Dark Triad traits during the hiring process. Additionally, implementing emotional intelligence and conflict management training programs is important to reduce negative behaviors. Promoting ethical leadership and creating clear organizational policies are essential to mitigate the effects of these traits. It is also recommended to provide psychological support to affected employees and conduct continuous monitoring to assess the impact of interventions.

## Data Availability

The manuscript did not include this statement as it is a systematic review of the literature and does not provide any data.

## Appendix A

**Table A1:** Characteristics and Results of the Studies.

Study/Author	Typology/Main Objective	Participants	Variables/Instruments	Main Findings	International Banking Institute (JBI)
(Kızıloğlu et al., 2024)	Design: Cross-sectional descriptive. Aim: To examine the relationship between dark personality traits and the Big Five personality traits in relation to workaholism.	n = 514 Sexo (w/b): 211/303 Age: >21 years	Workaholism: BWAS Dark triad: Adaptation of Single-Item Narcissism Scale for dark triad. Large Personality Cunco: Single items.	Narcissism (r = 0.28, *p* < 0.001), Machiavellianism (r = 0.20, *p* < 0.001), psychopathy (r = 0.024, *p* < 0.001), sadism (r = 0.20, *p* < 0.001), spitefulness (β = 0.11, *p* < 0.05) and neuroticism (r = 0.13, *p* < 0.01) were positively associated with workaholism. Extroversion (β = −0.19, *p* < 0.001) and openness (β = −0.16, *p* < 0.01) were negatively correlated with workaholism.	7/8
(Silvester et al., 2014)	Design: Cross-sectional descriptive. Aim: investigating the personality characteristics of political workers and their relationship to performance appraisals.	n = 1478 Etapa 1: 53 Etapa 2: 240	Political competences: PPQ Political Skills: PSI Personality: NEO-PI-R Machiavellianism: Machiavellianism IV.	Five key factors were identified in political skills: resilience (18.82% variance explained), politicking (11.68% variance), analytical skills (10.29% variance), representing people (7.91% variance) and relating to others (5.86% variance). Intra-class correlations (ICC) were moderately low (median = 0.12), indicating variability in ratings between politicians and between officials. Multilevel analyses showed adequate fit in both groups (CFI = 0.90, RMSEA = 0.04). Conscientiousness correlated positively with analytical skills (AS) and representation (RP), and negatively with politicking (PK) (*p* < 0.05). Neuroticism showed a negative correlation with resilience (RS) and analytical skills (AS) in both self-assessments and evaluations received. Political Ability correlated positively with all skills except politicking (*p* < 0.05). Conscientiousness and Political Ability positively predicted ratings received on RS, RP and RO, although extroversion was not significant in this respect. Machiavellianism was significantly associated only with the Politicization (PK) factor, while RS, RP and RO ratings were negatively affected by the presence of this trait.	8/8
(Simonet et al., 2018)	Design: Cross-sectional descriptive. Aim: investigating how interactions between dark triad personality traits, in combination with other personality traits, affect leadership performance	Nt = 1070 N1 = 285 Position: Lower-middle managers Sex (M/F): 199/86 N2: 120 Position: Senior managers Sex (M/F): 75/45 Average age: 37.7 years N3: 106 Position: Advanced recruits Average age: 29.8 years N4: 599	Dark Triad: HDS Big Five: HPI Leadership performance: Likert scale items.	The interaction between trait narcissism and antisocial tendencies showed a negative effect on leadership performance. In samples 1 and 4, leaders with high levels of both traits performed significantly lower (with a value β = −0.02, *p* < 0.05). The combination of narcissism and histrionic traits (excessive attention seeking) also had a negative impact on performance. In sample 2, leaders with high levels of these traits showed lower performance (β = −0.01, *p* < 0.05). Emotional stability moderated the effects of narcissism. In sample 1, leaders with high levels of narcissism and low emotional stability (low neuroticism) showed significantly lower performance (β = 0.01, *p* < 0.05). The strength of the effects of these traits varies by organizational context. In less regulated industries, such as the retail sector, the interactions between the Dark Triad traits and leadership performance were more pronounced. In contrast, in more regulated contexts, such as pharmaceuticals, these effects were not as significant. The combined effects of these traits suggest that leaders with high levels of multiple Dark Triad traits tend to experience greater performance detraction. Interactions were more evident in lower-ranking positions, underscoring that environment and role type significantly influence the effect of these traits.	8/8
(McKee et al., 2017)	Design: Cross-sectional descriptive. Aim: Explore whether followers’ personality characteristics (including Dark Triad and Big Five traits) relate to their preferences for leaders who exhibit certain “dark” behaviors.	n = 167 Age: >18 años Sex (g/b): 85/82	Dark Triad Personality: SD3 Big Five: BFI Dark leadership behaviors: Hare P-Scan	Psychopathy (r = 0.45, *p* < 0.01) and neuroticism trended positively with desire for dark leadership behaviors. Agreeableness (r = −0.34, *p* < 0.01) and conscientiousness (r = −0.36, *p* < 0.01) showed a significant negative relationship with desire for dark leadership behaviors. The Dark Triad predicted preference for dark leaders over and above factors such as age, gender and employment status (ΔR^2^ = 0.21, ΔF = 16.32, *p* < 0.001), confirming the hypothesis that dark traits in followers may influence their dark leadership preferences. The inclusion of Big Five traits in the regression did not contribute significant additional variance (ΔR^2^ = 0.02, ΔF = 0.96, *p* = 0.45), suggesting that the Dark Triad captures a key part of the variability in preference for leaders with dark traits, which is not explained by Big Five traits.	8/8
(Grover & Furnham, 2021)	Design: Cross-sectional descriptive. Aim: examine how the situational context (work vs. non-work) influences the expression of dark and general personality traits.	n = 903 Age: 34.2 (average) Sex (g/b): 508/395	Psychopathy: LSRP Narcissism: NPI-16 Machiavellianism: MPS Big Five: Mini-IPIP	Extraversion (M = 3.61) and responsibility (M = 4.20) were significantly higher in the work group than in the non-work group (*p* < 0.05). Neuroticism was significantly lower in the work group (M = 3.36) compared to the non-work group (M = 3.53) (*p* < 0.05). Machiavellianism was significantly lower in the work group (M = 2.85) compared to the non-work group (M = 3.01) (*p* = 0.002). Females showed significantly higher levels of agreeableness (M = 4.77) than males (M = 4.29) in the work group (*p* < 0.001).	8/8
(DeShong et al., 2017)	Design: Cross-sectional descriptive. Aim: investigating the relationship between Dark Triad personality traits (psychopathy, narcissism and Machiavellianism) and the Big Five personality dimensions (openness, responsibility, extraversion, agreeableness and neuroticism) in workers, and how these interactions affect job performance and workplace behavior.	n = 163 Age = 18–54 años Sex (g/b) = 117/46	Narcissism: FFNI Machiavellianism: MPS Psychopathy: EPA Big Five: NEO-PI-R Organizational Civic Behavior: OCB-C Unethical Behavior: UBS Workplace Deviance: WDS	Males scored significantly higher on Machiavellianism (t(156) = 2.17, *p* < 0.05) and on the Desire to Control subscale (t(153) = 2.10, *p* < 0.05), on psychopathy (t(105) = 2.14, *p* < 0.05) and on DD Psych (t(57.40) = 3.86, *p* < 0.001) and on the openness to ideas facet (t(155) = 5.48, *p* < 0.001). Females scored higher on kindness (t(133) = −2.23, *p* < 0.05) and on the subfactors sincerity (t(152) = −3.00, *p* < 0.01), altruism (t(153) = −2.03, *p* < 0.05), and anxiety (t(94.43) = −3.68, *p* < 0.001) and vulnerability (t(154) = −3.74, *p* < 0.001). Machiavellianism was negatively correlated with agreeableness (r = −0.49, *p* < 0.01). At the facet level, significant negative correlations with trustworthiness, sincerity and altruism were observed (r = −0.47 to −0.30, *p* < 0.01). Dark triad traits showed negative correlations with agreeableness (r = −0.56 for Machiavellianism and r = −0.72 for Psychopathy, both *p* < 0.01). Machiavellianism correlated negatively with conscientiousness (r = −0.30, *p* < 0.01) and the diligence and self-discipline subfactors (r = −0.34 and r = −0.26, *p* < 0.01).	6/8
(Wiens & Walker, 2019)	Design: Cross-sectional descriptive. Aim: Study 1—to investigate the relationships between bright personality traits (as measured by the CPI) and dark personality traits (as measured by the HDS) in a sample of managerial-level employees. Study 2—to extend the findings of Study 1 by examining the relationship between bright personality traits and dark personality traits as assessed by observers (supervisors), rather than relying solely on self-assessments.	N1: 262 N2: 113 Age 1: N/E Age 2: 49.7 years Sex (g/b) 1: 63/205 Sex (g/b) 2: 16/97	Bright Personality: CPI Dark Personality: HDS Critical Thinking Skill: Watson-Glaser Critical Thinking Appraisal	Psychopathy correlated negatively with Responsibility (r = −0.21, *p* < 0.05), empathy (r = −0.20, *p* < 0.05) and self-control (r = −0.10, *p* < 0.05, r = −0.10, *p* < 0.05, r = −0.10, *p* < 0.05). Machiavellianism correlated negatively with self-control (r = −0.21, *p* < 0.05), Responsibility (r = −0.19, *p* < 0.05), empathy (r = −0.30, *p* < 0.01) and sociability (r = −0.26, *p* < 0.01). Narcissism correlated negatively with self-control (r = −0.21, *p* < 0.01), empathy (r = −0.30, *p* < 0.01), responsibility (r = −0.36, *p* < 0.01) and sociability (r = −0.15, *p* < 0.05). Empathy has a significant negative beta coefficient (β) on the trait Excitable (−0.22, *p* < 0.05). Independence (In): Also shows a negative effect on Cautiousness (−0.15, *p* < 0.05). Good Impression (Gi): Shows a significant negative effect on Excitable (−0.26, *p* < 0.05) and Skeptical (−0.13, *p* < 0.05). Flexibility and Responsibility have a significant negative effect on the trait Boldness (−0.36, *p* < 0.05 and −0.21, *p* < 0.05, respectively). Empathy has a significant positive coefficient on the subclinical antisocial trait (0.22, *p* < 0.05).	6/8
(Harrell et al., 2024)	Design: Cross-sectional correlational (and descriptive). Aim: To investigate the relationship between the ‘Dark Core’ of personality, which includes traits such as Machiavellianism, narcissism and psychopathy, and the ‘General Factor of Personality’ (GFP), which represents positive personality traits in a sample of workers.	N1: 1066 N2: 1052 Age 1: 37.36 Age 2: 38.95 Sex (g/b) 1: 652/414 Sex (g/b) 2: 621/431	Dark Triad: SD3 General Personality Factor: HEXACO-100, IPIP-NEO-120 (sample 2 only)	A statistical analysis showed that the model connecting the personality traits (HEXACO and Dark Triad) fitted well, with a χ^2^ value (6749) = 25,553.13. This indicates that the model is adequate for the data, although some indicators suggest that it could be improved (CFI = 0.567 and TLI = 0.558 are low). When entering a model representing the General Personality Factor (GFP) and Dark Core, it was found that there was a strong negative relationship between them: −0.93 (*p* < 0.001). This means that as a person has more dark traits (such as narcissism), they tend to have fewer positive personality traits. In the HEXACO trait analysis, the general component (GFP) was found to explain 31% of the variability in personality traits, while the Dark Triad explains 57% of the variability in its traits. This shows that dark traits have a greater impact on the variability of personalities in this sample. When analyzing another sample (Sample 2), a different model was used and fitted well after changes: χ^2^ (538) = 4161.636, CFI = 0.858, TLI = 0.833. This means that the model fitted the new data well too. In Sample 2, the factors representing the Dark Core explained up to 85% of the variability in some measures, indicating that the dark traits are consistent and significant in this group as well. The correlations between GFP and Dark Core in Sample 2 range from −0.38 to −0.46. This reinforces the idea that there is a negative relationship between these traits in both samples. Machiavellianism: Negative correlation with Honesty-Humility (r = −0.58). Narcissism: Negative correlation with Agreeableness (r = −0.37). Psychopathy: Negative correlation with Honesty-Humility (r = −0.51).	7/8
(Shi et al., 2024)	Design: Cross-sectional correlational (and descriptive). Aim: To investigate the relationship between personality characteristics of supervisors in the context of the hotel industry with envy and abusive supervision.	n = 188 supervisor-subordinate pairs Age: >24 Sex (g/b): 149/88	Dispositional Supervisor Envy: Lange and Crusius Dispositional Malicious Envy Scale. Supervisor’s episodic envy of subordinate: Cohen-Charash and Mueller scale. Supervisor’s power dependence on subordinate: IteWee et al. Abusive supervision: Tepper scale. Only in study 2: Supervisor narcissism: Narcissism scale by Resick et al. Supervisor’s neuroticism: Sato Neuroticism Scale.	Study 1: A significant positive correlation was found between dispositional envy and episodic envy (r = 0.59, *p* < 0.001). Dispositional envy was also positively correlated with perceived abusive supervision (r = 0.19, *p* < 0.01). Forty per cent of the variance in episodic supervisor envy was due to dyad differences. Dispositional envy predicts episodic envy (estimate = 0.49, *p* < 0.001) and perceived abusive supervision (estimate = 0.12, *p* < 0.05). No evidence was found that episodic envy mediated between dispositional envy and abusive supervision. Power dependence affects the relationship between dispositional envy and episodic envy, being stronger when dependence is low (estimate = 0.70, *p* < 0.001). Study 2: Correlations confirmed similar patterns, showing a positive relationship between dispositional envy and episodic envy (estimate = 0.48, *p* < 0.001) and perceived abusive supervision (estimate = 0.52, *p* < 0.001). Episodic envy strongly predicts perceived abusive supervision (estimate = 0.93, *p* < 0.001). Episodic envy fully mediates the relationship between dispositional envy and abusive supervision, supporting mediation. The relationship between dispositional envy and episodic envy is stronger in supervisors with high levels of narcissism (estimate = 0.80, *p* < 0.001). The relationship between dispositional envy and episodic envy was also found to be stronger in supervisors with high levels of neuroticism (estimate = 1.03, *p* < 0.001).	8/8
(Schattke & Marion-Jetten, 2022)	Design: Cross-sectional correlational (and descriptive). Aim 1: To explore the nomological network of dominance, prestige and leadership in relation to narcissism, Machiavellianism and psychopathy, as well as obsessive compulsive behavior and pro-social behavior (OCB). Study 2: To replicate the nomological network established in Study 1 with a population of leaders, investigate associations with transformational and transactional leadership styles, and test the mediation hypothesis using trajectory analysis.	N1: 151 N2: 361 Age 1: 21–64 Age 2: 40.61 Sex (g/b) 1: 81/64 Sex (g/b) 2: 180/181	Explicit power motives: DoPL Dark Triad: The Dirty Dozen Scale Counterproductive Work Behavior (CWB): CWB Checklist. Constructive Organisational Behavior (OCB): OCB Checklist. Leadership styles: Multifactor Leadership Questionnaire (Study 2).	Study 1: Dominance was positively correlated with all three dark triad traits (narcissism r = 0.59, *p* < 0.001; Machiavellianism r = 0.50, *p* < 0.001; psychopathy r = 0.01). Prestige strongly correlated with narcissism (r = 0.30, *p* < 0.01) and weakly with Machiavellianism (r = 0.13); it did not correlate with psychopathy. Leadership moderately correlated with narcissism (r = 0.26, *p* < 0.01). CWB (counterproductive work behavior) positively correlated with dominance (r = 0.266, *p* < 0.01); unexpectedly, it also correlated with OCB (organizational citizenship behavior) (r = 0.21, *p* < 0.05). Study 2: The correlations of dominance, prestige, and leadership with the dark triad were similar to those in Study 1. Dominance was positively correlated with transactional leadership (r = 0.42, *p* < 0.001). Prestige and leadership were more strongly correlated with both leadership styles, with prestige being more strongly correlated with transactional leadership (r = 0.30, *p* < 0.001). All power motives significantly predicted transformational leadership, with a negative coefficient for dominance and positive ones for prestige and leadership. Transformational leadership significantly predicted both OCB (r = 0.39, *p* < 0.001) and CWB (r = −0.16, *p* < 0.05).	6/8
(Furnham & Crump, 2014)	Design: Cross-sectional correlational (and descriptive). Aim: Examine which specific facets of the Big Five personality traits are associated with the trait of boldness or narcissism (NPD) in the workplace.	n = 6957 Sex (g/b): 5464/1493 Age: 23–65 años Position: Middle—Senior Manager.	Big five: NEO-PI-R Boldness/Narcissism:HDS	Boldness/Narcissism: Boldness has a negative correlation with Neuroticism (−0.13) and Agreeableness (−0.24); and a positive correlation with Extraversion (0.30), Openness to Experience (0.13), and Conscientiousness (0.21). Agreeableness has a strong negative effect on boldness (β = −0.28, *p* < 0.001). Extraversion and Conscientiousness have moderate positive effects on boldness (Extraversion β = 0.23, *p* < 0.001; Conscientiousness β = 0.16, *p* < 0.001). Demographic variables like gender and age did not influence boldness. The model explains 20% of the variance in boldness (Adj. R^2^ = 0.20). Neuroticism: Vulnerability (N6) is negatively related to boldness (−0.28), and Hostility (N2) has a positive relationship (0.16). Extraversion: Assertiveness (E3) has a significant positive relationship with boldness (0.27). Agreeableness: Modesty (A5) is strongly negatively related to boldness (−0.37). Conscientiousness: Competence (C1) is positively related to boldness (0.26).	8/8
(Paleczek et al., 2018)	Design: Cross-sectional correlational (and descriptive). Aim: Explore how the traits of the Dark Triad, along with the traits of the Big Five, are related to specific variables of job success and job satisfaction.	n = 287 Age: 37.74 Sex (g/b): 150/137 Position: Leaders/Non-leaders	Big Five: NEO-PI-R Narcissism: NPI-R Machiavellianism: MACH-IV Psychopathy: LSRP Objective career success: Annual salary and binary indicator of leadership position (yes/no) Job satisfaction: Fahrenberg’s life satisfaction questionnaire.	The traits of the Dark Triad (narcissism, Machiavellianism, and psychopathy) are positively correlated with each other, with a particularly strong association between Machiavellianism and psychopathy (r = 0.63, *p* < 0.01). Psychopathy showed a high negative correlation with agreeableness (r = −0.65, *p* < 0.01), which represents the most significant correlation between a dark trait and a positive one. The inclusion of the Dark Triad improved the prediction of salary (ΔR^2^ = 0.02, *p* < 0.05) and leadership position (ΔR^2^ = 0.04, *p* < 0.05). Narcissism was the only trait that positively predicted both salary (β = 0.16, *p* < 0.05) and leadership position (Exp(B) = 7.36, *p* < 0.01). Psychopathy had a negative effect on salary (β = −0.19, *p* < 0.05), while Machiavellianism had no significant effect on objective career success. Using only the Big Five traits, the model correctly classified 70.8% of participants in terms of whether they had a leadership position. When adding the Dark Triad traits, classification accuracy increased to 74.3%. The personality traits (Big Five and Dark Triad) explained 20% of the variance in job satisfaction, 13% in income satisfaction, 6% in salary, and 13% in leadership position.	8/8
(Ramos-Villagrasa et al., 2020)	Design: Cross-sectional correlational (and descriptive). Aim: Research how personality (Dark Triad and Big Five) influences adaptive work performance.	n = 613 Age: 18–70 Sex (g/b): 282/331	Big Five: NEO-FFI Dark Triad: Dark Triad at Work, adapted Adaptive performance: Scale developed by Marques-Quinteiro, adapted	All the Big Five traits showed significant correlations with each other, except for Neuroticism with Openness to Experience (r = 0.04, *p* = 0.324) and Openness to Experience with Agreeableness (r = 0.06, *p* = 0.190). Neuroticism showed significant negative associations with Extraversion (r = −0.33, *p* < 0.001), Agreeableness (r = −0.26, *p* < 0.001), and Conscientiousness (r = −0.40, *p* < 0.001). The relationship between Psychopathy and Sadism was high (r = 0.67, *p* < 0.001), while Narcissism and Machiavellianism showed no significant association (r = 0.02, *p* = 0.576). Adaptive performance had significant positive associations with Extraversion (r = 0.25, *p* < 0.001), Openness (r = 0.21, *p* < 0.001), Agreeableness (r = 0.13, *p* = 0.002), Conscientiousness (r = −0.30, *p* < 0.001), and Narcissism (r = 0.16, *p* < 0.001). Neuroticism (r = −0.29, *p* < 0.001), Machiavellianism (r = −0.19, *p* < 0.001), Psychopathy (r = −0.24, *p* < 0.001), and Sadism (r = −0.17, *p* < 0.001) showed significant negative correlations with adaptive performance. Socio-demographic variables such as gender, age, and work experience showed no significant associations with adaptive performance. Control variables did not contribute significantly to explaining adaptive performance, with an R^2^ = 0.003, *p* = 0.677. When including the Big Five personality traits, 16.0% of the variance in adaptive performance was explained (ΔR^2^ = 0.159, *p* < 0.001), with Neuroticism (β = −0.165, *p* = 0.001), Openness (β = 0.186, *p* < 0.001), and Conscientiousness (β = 0.208, *p* < 0.001) being significant predictors. The inclusion of the Dark Tetrad increased the explained variance to 20.2% (ΔR^2^ = 0.184, *p* < 0.001), with Neuroticism (β = −0.127, *p* = 0.010), Openness (β = 0.155, *p* < 0.001), Conscientiousness (β = 0.164, *p* < 0.001), Narcissism (β = 0.134, *p* = 0.002), and Psychopathy (β = −0.147, *p* = 0.005) as significant predictors. The inclusion of Sadism in the model did not increase the explained variance (ΔR^2^ = −0.001, *p* = 0.541).	8/8
(Fernández-del-Río et al., 2020)	Design: Cross-sectional correlational (and descriptive). Aim: Explore the incremental validity of dark personality, conceptualized as (low) Honesty-Humility, narcissism, Machiavellianism, psychopathy, and sadism, in predicting three dimensions of job performance: Task Performance, Organizational Citizenship Behavior, and Counterproductive Work Behavior.	n = 613 Age: 38.78 Sex (g/b): 330/283	Big Five: NEO-FFI Honesty-Humility: HEXACO-PI-R Dark Tetrad: Dark Tetrad at Work Job Performance: Individual Job Performance (Adapted to Spanish).	The Dark Triad showed negative correlations with agreeableness (average |r| = 0.27). The correlations between Honesty-Humility and the traits of the Dark Tetrad were moderate (average |r| = 0.35). Psychopathy and sadism showed the strongest correlation with each other (r = 0.67, *p* < 0.001). The dimensions of job performance showed low to moderate correlations with the Dark Tetrad (average |r| = 0.22), with Counterproductive Work Behavior (CWB) being the most strongly associated (r = 0.26). The inclusion of Honesty-Humility did not increase the explained variance in Task Performance (TP) and Contextual Performance (CP) (ΔR^2^ = 0.00), but did increase it in CWB (ΔR^2^ = 0.03, *p* < 0.001). The incorporation of the Dark Tetrad increased the explained variance in TP (ΔR^2^ = 0.06, *p* < 0.001), CP (ΔR^2^ = 0.11, *p* < 0.001), and CWB (ΔR^2^ = 0.04, *p* < 0.001). The inclusion of sadism increased the explained variance in TP (ΔR^2^ = 0.01, *p* = 0.029) and CWB (ΔR^2^ = 0.01, *p* = 0.004), but did not increase it in CP. For task performance (specific tasks), the traits that predicted higher scores were: • Conscientiousness (β = 0.37, *p* < 0.001) • Narcissism (β = 0.23, *p* < 0.001) • Machiavellianism (β = 0.10, *p* = 0.025) For contextual performance (behavior in the environment), the positive predictors were: • Openness (β = 0.17, *p* < 0.001) • Conscientiousness (β = 0.22, *p* < 0.001) • Narcissism (β = 0.34, *p* < 0.001) • Machiavellianism had a negative association (β = −0.18, *p* < 0.001). Regarding CWB, neuroticism (β = 0.12, *p* = 0.011) and sadism (β = 0.16, *p* = 0.004) were positively associated, while Honesty-Humility had a negative association (β = −0.13, *p* = 0.009).	8/8
(Furnham & Treglown, 2021)	Design: Cross-sectional correlational (and descriptive). Aim: Understand how high-potential personality traits and the Dark Triad, along with work engagement, relate to perceptions of job success in participants.	n = 290 Sex (g/b): 109/181 Age: 34.1	High-potential personality traits: HPTI Inventory Dark Triad: Dirty Dozen Work engagement: UWES Perceived job success: Job success questionnaire	Women scored significantly higher in Conscientiousness (HPTI), Vigor (*p* = 0.014), and Dedication (*p* = 0.048) in work engagement. There were no significant gender differences in the Dark Triad traits. Subjective success was significantly and positively correlated with Courage, Conscientiousness, and Tolerance for Ambiguity (HPTI), and with Narcissism (r > 0.60) in the work engagement scales. Curiosity was the only trait in the HPTI that negatively correlated with subjective success. Courage and Psychopathy (positively), and Neuroticism and Tolerance for Ambiguity (negatively) predicted levels of Vigor, explaining 28.9% of the variance. Narcissism was the only significant predictor for Dedication and Absorption, explaining 30.1% and 26.3% of the variance, respectively. Neuroticism (negatively), and Courage and Narcissism (positively) predicted subjective success, explaining 23.8% of the variance. Work engagement, specifically Vigor and Dedication, increased the explanatory power of the model by 25.9%, reaching a total of 52.8%. Work engagement (comprised of Vigor, Dedication, and Absorption) was shown to have a mediating effect on the relationship between Conscientiousness and Narcissism and subjective success (ß indirect for Conscientiousness = 0.12, *p* < 0.001; ß for Narcissism = 0.26, *p* < 0.001). The model showed an acceptable fit overall (CFI = 0.97, TLI = 0.95), with some fit indices such as RMSEA indicating a moderate fit.	7/8
